# Transcatheter dilation and stenting of the modified blalock-taussig shunt in cyanotic heart diseases: points to consider. A single-center experience

**DOI:** 10.3389/fcvm.2024.1445987

**Published:** 2024-08-08

**Authors:** Nathalie Mini, Peter A. Zartner, Martin B. E. Schneider

**Affiliations:** Cardiac Catheterization Laboratory, Department of Pediatric Cardiology, Hospital University of Bonn, Bonn, Germany

**Keywords:** mBT shunt, shunt stenting, shunt stenosis, HLHS, hypoplastic left heart syndrome, shunt obstruction, pulmonary atresia

## Abstract

**Background:**

Our study focuses on the technique and results of interventional dilation and stenting of the modified Blalock–Taussig shunt (MBTS) performed in our center, providing a comprehensive review of our practice over the past 4 years.

**Methods:**

42 catheter interventions on MBTS performed on 32 patients between January 2020 and May 2024 included 11 balloon dilatations and 31 stenting procedures. They were analyzed retrospectively. We documented early and late complications, the need for in-shunt reintervention or surgical revision, in-stent thrombotic events, and sudden death.

**Results:**

The median age, weight, body surface area, and procedural radiation time at the time of the intervention were 100 days (15–870 days), 5 kg (2.6–12 kg), 0.3 m^2^ (0.19–0.54 m^2^), and 12 min (7–28 min). Four interventions were performed as an emergency in three patients, of which three were performed with ECMO support. The interventions were semi-emergent for severe hypoxia in 22 patients and elective for mild desaturation in the rest. All interventions were successfully performed without any intervention-related complications or death. Eighty-two percent of the shunt dilations led to saturation improvement. Of the 31 shunt stentings performed in 26 patients, saturation improvement was documented in 97% of the cases. The late complication with stent thrombosis was documented in one patient. In two patients, sudden death and cardiac decompensation with the need for resuscitation were documented.

**Conclusion:**

MBTS interventions are effective in emergent and semi-emergent situations with severe hypoxia. While serial balloon dilatations improved the luminal diameter in clipped MBTS, most patients needed stenting as a definitive procedure. In some cases, enlargement of the shunt via stenting may be an alternative to the surgical shunt revision to accommodate the shunt size and weight and delay the subsequent operation when there are contraindications. Dual antiplatelet therapy is strongly recommended to reduce thrombotic events, especially in shunts with more than one stent and those that need reinterventions.

## Introduction

The modified Blalock–Taussig shunt (MBTS) is a synthetic tube graft of polytetrafluoroethylene (PTEF; Gore-Tex). The shunt is created between the subclavian artery and the branch pulmonary artery to provide adequate pulmonary perfusion in patients with cyanotic heart failure, like hypoplastic left heart syndrome (HLHS), who have undergone Norwood palliation and in those with pulmonary atresia (PA), in whom interventional palliation could be difficult. However, MBTS palliation is associated with high morbidity and mortality (1).

Over time, many shunts will develop some degree of stenosis due to shunt intima proliferation, thrombosis, and, in some cases, shunt torsion. Wells et al. reported that 34% of shunts showed a luminal narrowing during elective shunt takedown, and 0.2% of patients had >50% stenosis of the shunt (2). Shunt obstruction, shunt stenosis, and thrombosis can occur early or late after the operation, ranging in severity from mild to severe to life-threatening. In such cases, an emergent intervention is required to recanalize the shunt and regain the shunt's patency and pulmonary perfusion to save the patient's life. Many studies about the MBTS have been conducted for a better understanding of its physiology and to prove the safety and efficacy of the intervention on it (3–6). At the same time, intervention-related complications like cardiac tamponade, catheter-induced transient complete atrioventricular block, and massive thrombo-embolic stroke were reported (4), as well as arrhythmias and damage of the vascular access (5).

In this study, we aim to review our experience in shunt intervention, the intervention technique, and the outcome of shunt stenting in our cohort and to focus on the essential points we have learned regarding intervention on MBTS.

## Patients and methods

Between January 2020 and May 2024, we retrospectively recruited 42 interventions in 32 patients with MBTS stenosis in our catheterization laboratory. Of these, 18 had HLHS, 10 had pulmonary atresia, and 4 had tricuspid atresia.

The shunt size was 3.5 mm in 18 patients, 4 mm in 11, 5 mm in 1, and 6 mm in 1. The interventions were performed as emergency procedures in four patients, semi-emergent for severe hypoxia in 22 patients and elective for mild desaturation in the rest. An angiographic examination of the shunt and the pulmonary branches was performed on all patients.

Activated clotting time (ACT) was examined at the time of catheterization (the aimed value of ACT ranges between 160 and 180 s), and 100 units/kg heparin was substituted.

Saturation before and after the intervention, early and late intervention-related complications, the need for ECMO, the need for reintervention in the shunt stent, and early and late death were documented. Since there was no evidence of neurologic events, they were clinically evaluated without planning any elective CT or MRI. An echocardiogram was used 6, 24, and 48 h after the intervention to exclude pericardiac effusion.

## Statistical analysis

All statistical analyses were performed using SPSS version 22. Continuous variables were reported as median ± standard deviation (SD), and categorical variables as count (percentage).

## Ethical statement

The study complies with the Declaration of Helsinki (as revised in 2013). As this retrospective study used previously available institutional clinical records, presented completely anonymized data, and did not impact patient management, no ethical committee approval was required. Parental consent for the shunt intervention was obtained for all interventions, including the emergent and semi-emergent procedures needed in 62% of the cohort.

## Intervention description

All interventions were performed under sedation by an experienced anesthesiologist. The exception was one patient who was resuscitated and intubated at the ICU for a suboccluded shunt. Parental consent was obtained before the interventions. Heparin 100 units/kg was administered as a bolus. ACT was measured, and an additional heparin bolus was given based on the ACT that was maintained between 180 and 200 s.

A 4-French (F) sheath (Terumo, Radifocus®, Introducer II) was introduced into the femoral artery. Aortic arch angiogram using a 4F angiographic catheter was used to demonstrate the shunt and the pulmonary arteries. The subclavian artery was then accessed with a 4 F end-hole catheter (usually Terumo®, Radifocus®, Glidecath™, or Cobra C1). The tip of the catheter was then positioned at the aortic side of the shunt.

### Shunt stenting

In the case of stenting, we introduced a 4 F long sheath (Flexor® Check-Flo® Introducer, 45 cm) into the TBC/subclavian artery. After that, ACT was measured again, and the heparin dose was adapted based on ACT. The shunt was then accessed using a 0.014-inch wire (such as WIZDOM™ Steerable Guidewire-Cordis or Mailman™ Guidewire, which can provide satisfactory balloon stability). The wire was positioned at the lower lobe of the branch pulmonary artery. In most cases, we delivered the stent into the shunt without pre-dilatation. After angiography ensured shunt patency, adequate pulmonary perfusion and excluded dissection, thrombosis, and stent malposition, the balloon and the guidewire were removed. The pericardial and pleural effusions were excluded based on echocardiograms.

We used the long sheath in stenting to ensure the stability of the wire and the stent, position the stent in the shunt while performing angiography using the side port of the long sheath, and prevent any stent dislocation by facilitating the removal of the stent balloon by pushing the long sheath while retrieving the balloon through the sheath.

**Figure 1:**
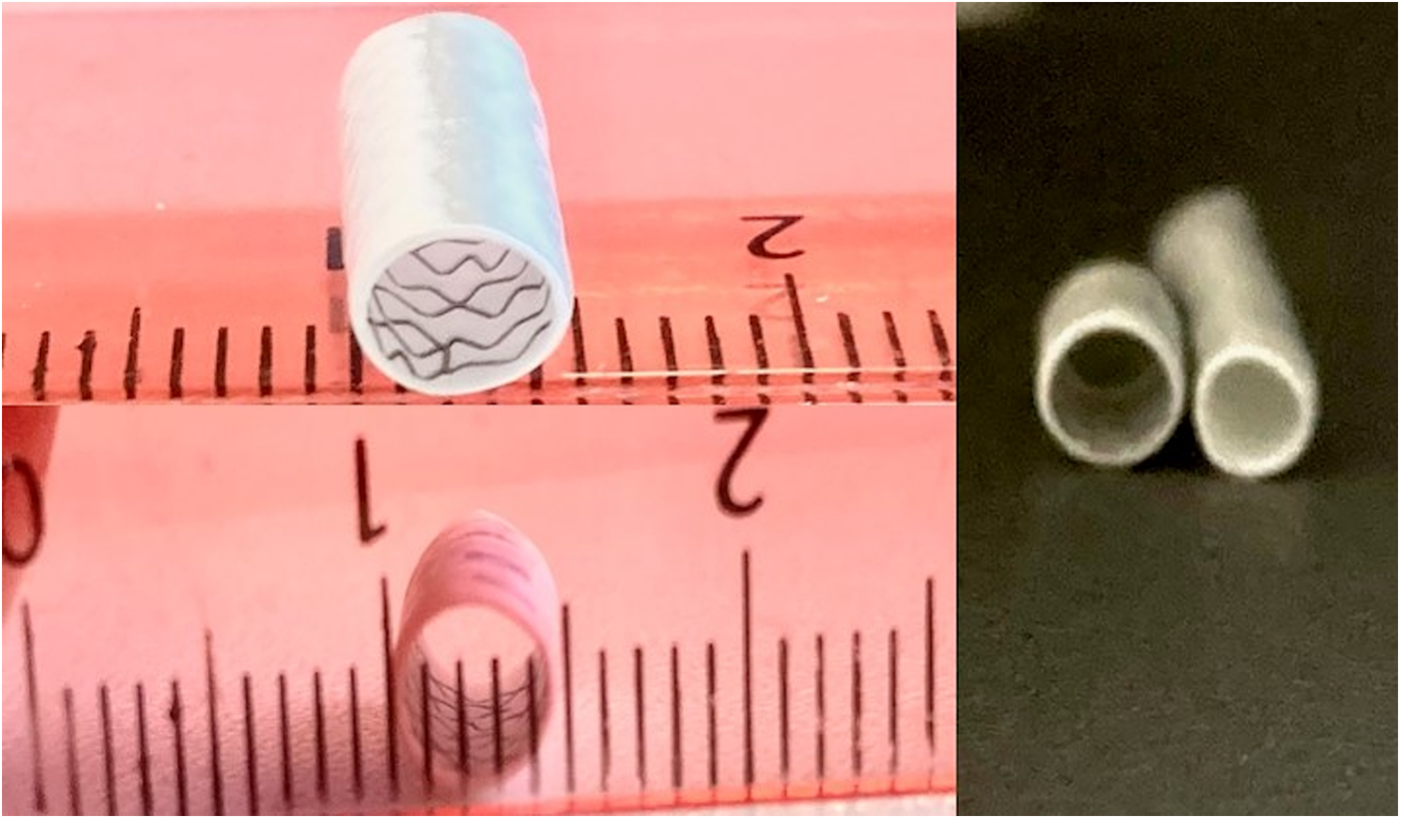
Demonstration of the shunt before and after stenting. (**A**) 3.5 mm shunt before and after stenting with a coronary stent of 4 mm, the shunt stent was dilated to a diameter of 4.5 mm.

In small children weighing under 4 kg, the stent size should equal the shunt size to prevent secondary pulmonary overcirculation. In patients weighing >4 kg who have contraindications for surgery in the usual time (age of 5–6 months) and will need the shunt longer than others, the stent size could be 1 mm more than the shunt size (Figure 1).

### Dilation of the shunt

**Figure 2:**
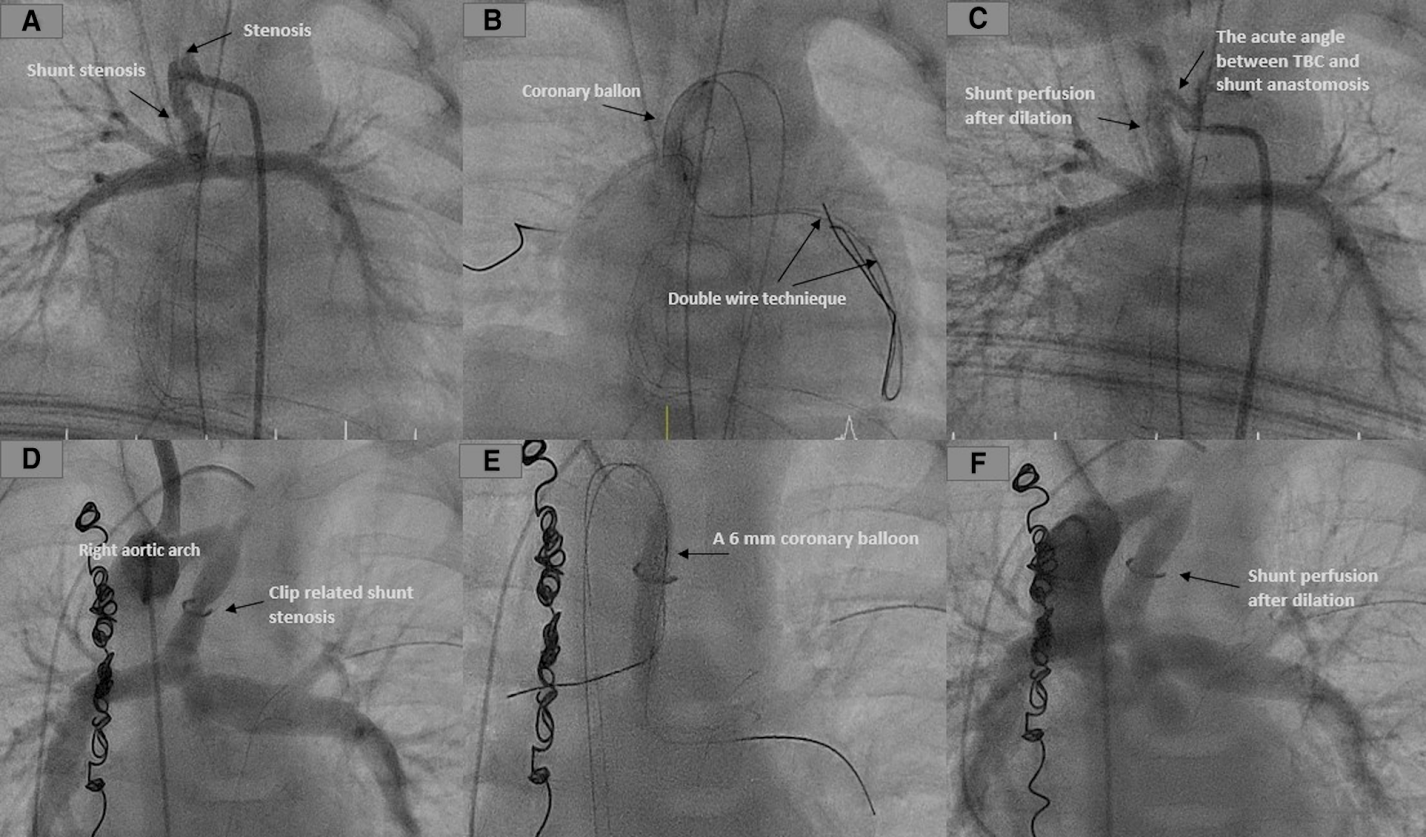
Dilation of shunt stenosis. (**A–C**) Dilation of the shunt stenosis in a patient with HLHS 2 days after the Norwood palliation due to desaturation. The acute angle between TBC and shunt anastomosis made crossing the shunt difficult. A double-wire technique was used to stabilize the introduction of the catheter and the balloon. The shunt was dilated with a coronary balloon, and there was significant saturation improvement. (**D–F**) Demonstrate the Dilation of the 6 mm clipped shunt in a patient with PA and congenitally corrected transposition of the great arteries (ccTGA). The Dilation was performed directly after the operation due to desaturation. The shunt was crossed using two wires and dilated to 6 mm with significant saturation improvement.

In the case of dilating the clipped shunt without stenting, we did not require a long sheath for the intervention. However, when the shunt was challenging to access because of torsion or atypical shunt connection to the TBC, a long sheath was required to provide more stability to the used materials. We accessed the shunt using a 0.035-inch GLIDEWIRE® Hydrophilic Coated Guidewire, which should be exchanged for a 0.014-inch coronary wire to accommodate the coronary balloon. In the latter case, we preferred to use the Mailman™ Guidewire from Boston Scientific, which provides satisfactory stability for the balloon (Figure 2).

The balloon size should be equal to the shunt diameter. A coronary balloon (like the NC EMERGE™ PTCA Dilatation Catheter) was then advanced over the 0.014-inch wire to the shunt, and the shunt clip was dilated. After that, the intervention result was ensured with angiography and echocardiography. The time of balloon-related shunt occlusion during the balloon inflation should be limited to a few seconds. At the same time, an experienced anesthesiologist must carefully monitor the vital and hemodynamic parameters.

Heparin 400 units/kg/d was administered for 48 h after the intervention. Aspirin 2–3 mg/kg/d was our choice as antiplatelet therapy until the subsequent surgery.

## Results

**Figure 3:**
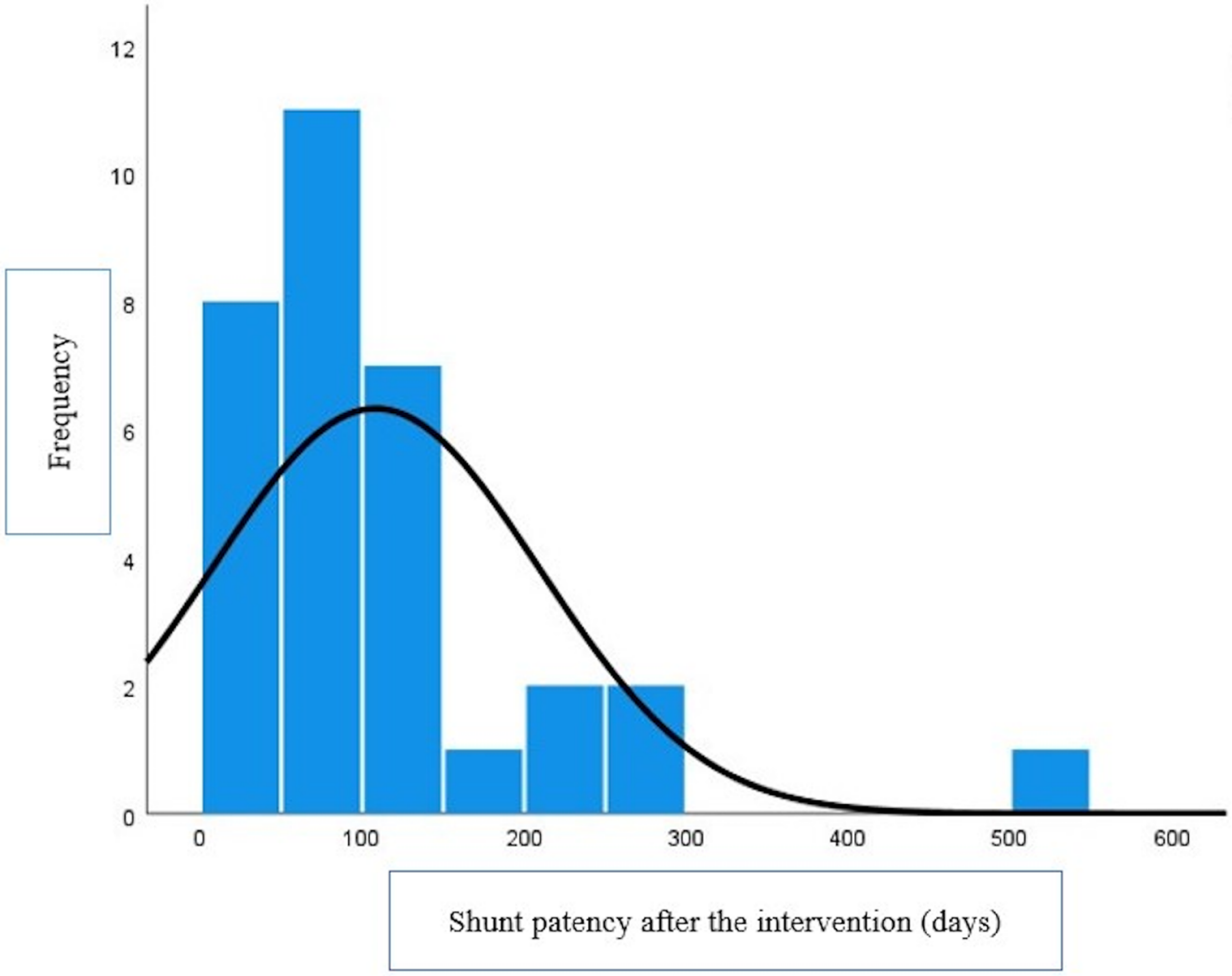
Histogram for shunt patency after intervention in 32 patients.

The median weight, age, BSA, and radiation time were 5 kg (2.6–12 kg), 100 days (15–870 days), 0.3 m^2^ (0.19–0.54 m^2^), and 12 min (7–28 min). The median stent patency was 79 (1–500 days) (Figure 3).

There was no intervention-related complication or death. No pericardiac or pleural effusion was documented 24, 48, and 72 h after the intervention.

### Emergency interventions

Four emergency interventions were needed due to acute shunt obstruction in three patients, twice in a same patient.

**Figure 4:**
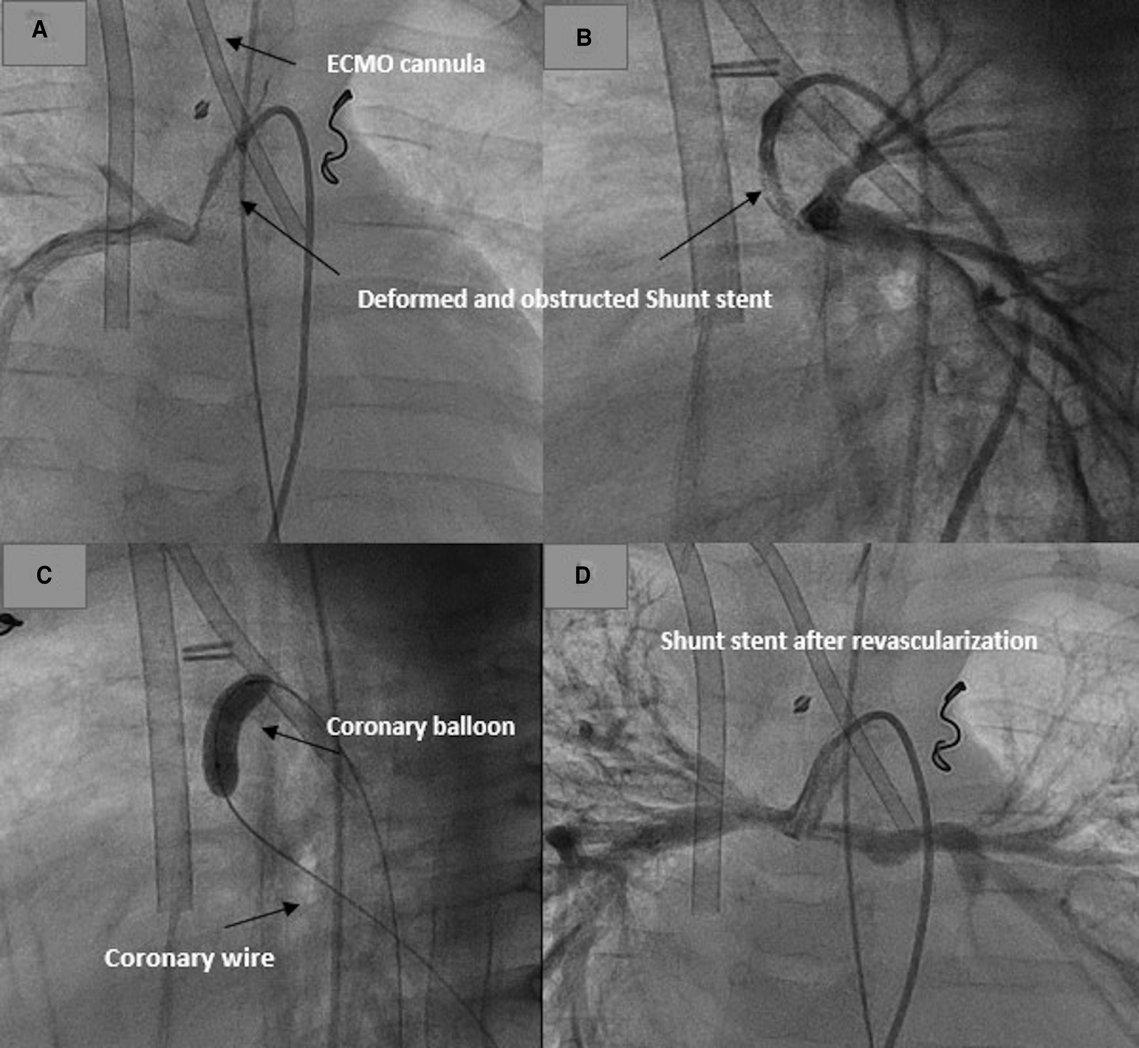
Revascularization of a deformed shunt stent after resuscitation in a patient on ECMO. (**A,B**) Deformed stent in AP and lateral projections. (**C**) Recanalization and dilatation of the stent. (**D**) Shunt and pulmonary perfusion after shunt revascularization.

The first patient with HLHS needed a shunt stent due to shunt stenosis and underdevelopment of the right or the left branch pulmonary artery. He was referred to palliative therapy. Sixty days after the shunt stenting, he had to be resuscitated during his hospital stay due to hemodynamic deterioration after a crying attack. Burst massage during resuscitation led to stent deformation. The stent was completely compromised with no perfusion. The shunt revascularization was performed while the patient was on ECMO (Figure 4). The ECMO was decannulated 24 h after stenting.

**Figure 5:**
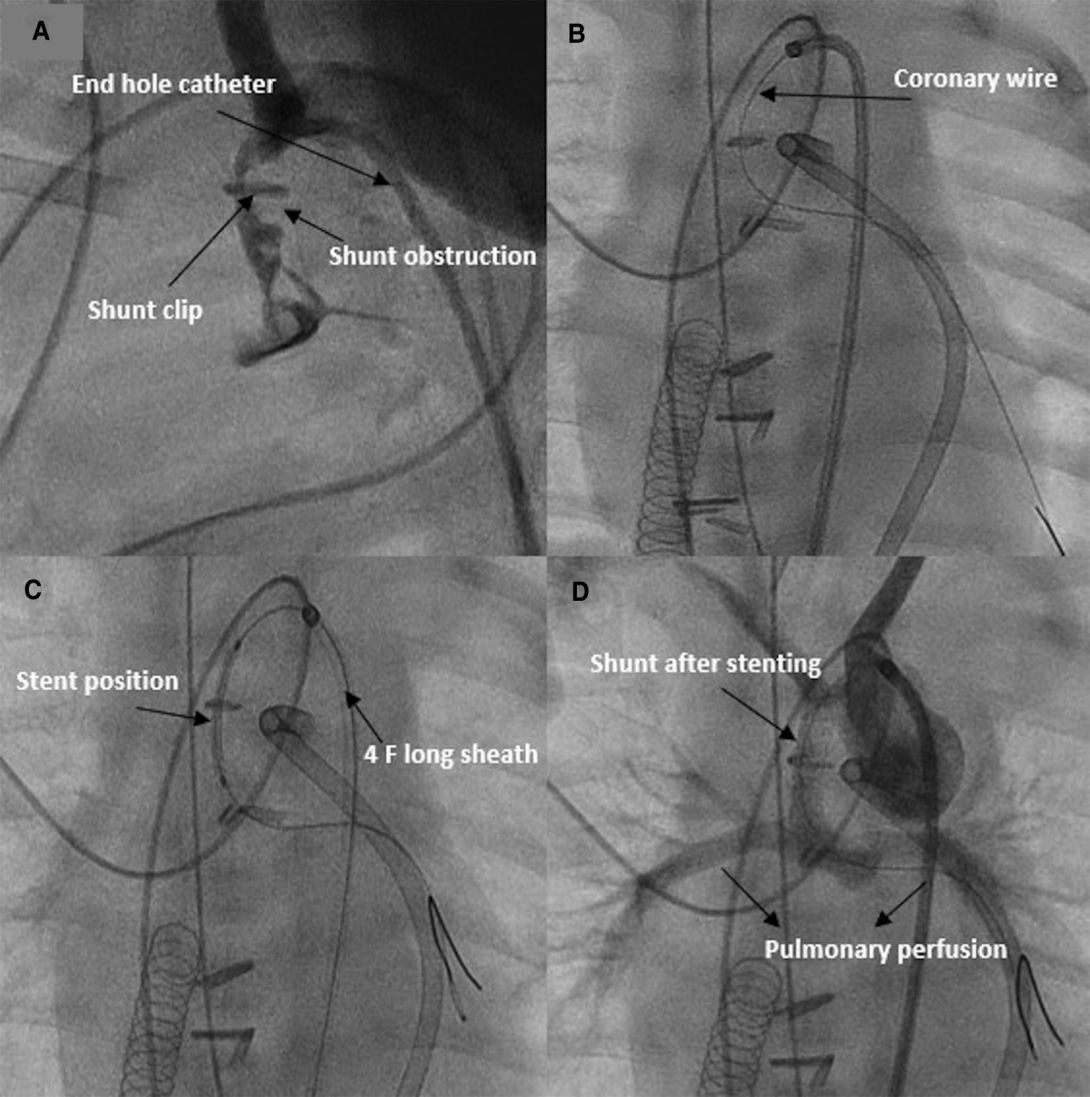
Revascularization and stenting of obstructed shunt postoperative. (**A,B**) Demonstrating the thrombosed shunt postoperatively in AP and lateral projection. (**C**) Recanalize the shunt with coronary wire and introduce the stent into the shunt. (**D**) Shunt and pulmonary perfusion after stenting.

The second patient (HLHS) had an impaired thrombophilia diagnostic, which led postoperatively to multiple thrombotic events, including multiple cerebral thromboses and infarcts and thrombotic occlusion of the brachiocephalic vein. Stenting of the obstructed brachiocephalic vein with the need for redilation by acute thrombosis was required. The patient was resuscitated by acute obstruction of the shunt postoperatively, and the shunt stenting was performed by ECMO standby in the hemodynamically unstable child, who needed a high dose of catecholamine (Figure 5). Despite the continuous intravenous heparin, a new in-stent intervention was required due to thrombosis of the shunt stent and the pulmonary arteries, which occurred 30 days after stenting.

**Figure 6:**
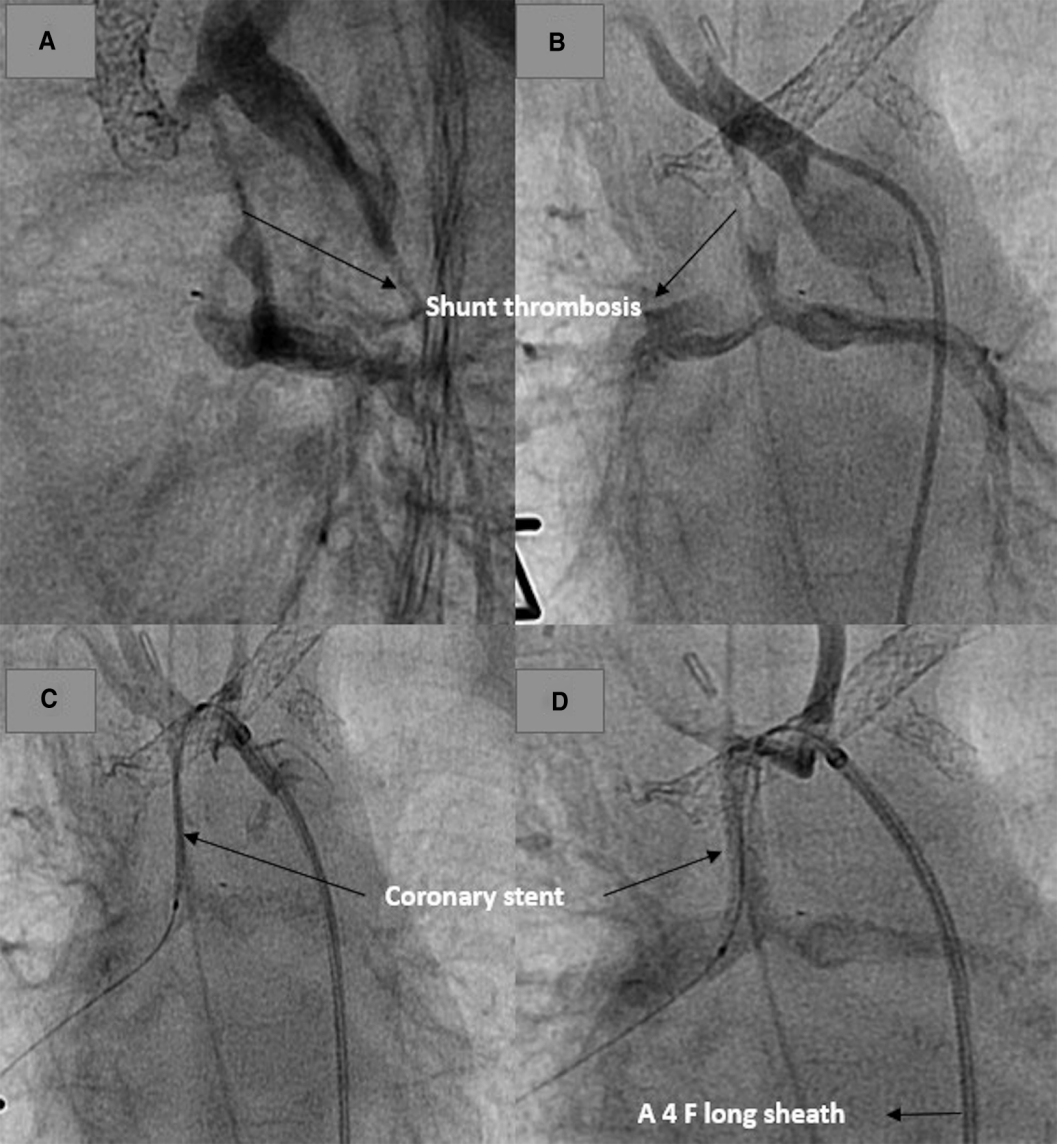
Revascularization of an obstructed shunt in a patient on ECMO. (**A,B**) demonstrate the thrombosed shunt with the clip. (**C**) Stent position after recanalization of the shunt. (**C**) Shunt and pulmonary perfusion after stenting.

The third patient (HLHS, Norwood Palliation) had an acute shunt obstruction postoperatively and needed ECMO for survival. The interventional shunt revascularization was performed while the patient was on ECMO (Figure 6). The shunt was revised after 24 h due to an ECMO-related thrombotic event. The patient died postoperatively on ECMO.

### Shunt dilation

Eleven balloon dilatations included seven procedures in clipped MBTS in six patients, one procedure in a shunt without a clip and three to balloon dilate a previously stented shunt. The shunt size was 3.5 mm in 2 patients, 4 mm in 2, 5, and 6 mm in 1. The weight at the intervention was 2.6, 2.7, 2.8, 3.9,4.4, 8.6, and 11 kg, respectively. The balloon size used for dilation was equal to the shunt size. In one patient weighing 2.6 kg, the clip of the 3.5 mm shunt was dilated first with 3 mm to avoid pulmonary overcirculation. He needed a second intervention to dilate the clip with a 3.5 mm balloon to accommodate the shunt size based on weight.

We reported the same condition in the second patient with a clip in a 4 mm shunt, who needed two separated shunt dilations with balloon sizes of 3.5 mm and 4 mm, respectively.

Shunt stent dilation was reported in three patients. In the first one, the stent had to be dilated to accommodate the shunt size and weight. In the second one, the obstructed shunt stent was recanalized. In the third patient, the wholly deformed stent was recanalized after resuscitation in the second patient, who was on ECMO.

All shunt and shunt stent dilations were performed without intervention-related complications.

The dilation of shunt clips and stents effectively improved the saturation in 9 of 10 interventions. In one patient with pulmonary atresia and hypoplastic right pulmonary artery, the dilation of the shunt (4 mm) was inadequate to improve the saturation. Two weeks later, the patient needed surgical shunt revision to a 5 mm shunt; then, the shunt in this patient had to be exchanged for a Sano shunt.

There were no shunt-related late complications.

### Shunt stenting

In 26 patients, 31 separate interventions with shunt stents were needed. Fifteen patients had HLHS, of whom 14 were in Stage I palliation and one had Glenn anastomosis and MBTS; four patients had tricuspid atresia, and the rest had pulmonary atresia with shunt palliation.

The indication of the shunt stent was shunt stenosis with desaturation in 23 interventions and acute shunt obstruction in 2, with the need for resuscitation and ECMO for survival. In 1 patient with MBTS in the left pulmonary artery and Glenn anastomosis in the right pulmonary artery, the indication of stenting was first to treat the shunt stenosis and second to separate the two circulations using a covered stent. One patient needed two interventions for stenting due to shunt stenosis in the first one and new stenosis distally of the first stent in the second one. Three patients needed a larger stent to delay the surgery due to hypoplastic branch pulmonary arteries at the time of operation.

In 26 interventions, the stents were coronary (1 BeGraft covered coronary stent, 4 Pro Kinetic Energy Biotronik, 3 Scaffold Magmaris stents and 18 Integrity stents).

Three interventions with three Magmaris® coronary resorbable magnesium scaffold stents were performed in 2 patients. One of them had pulmonary atresia and severe hypoplasia of the native branch pulmonary arteries with MAPCAs and required two interventions for stenting of the RPA with Magmaris stents. The interval between the two stents was three months. The need for reintervention was due to restenosis in the RPA and pulmonary side of the shunt. The pulmonary side of the shunt had to be included in stenting to ensure coverage of the proximal side of the RPA and to stent the distal stenosis of the shunt. The second patient had distal stenosis of the shunt with severe proximal LPA stenosis, and the shunt was included in stenting to cover the proximal stenosis of LPA.

The sirolimus level was checked 8 h, 24 h, and 48 h after stenting and was under 5 ng/ml without any documented side effects.

All shunt stentings were successfully performed without intervention-related complications or deaths. Follow-up was done in all patients except for the patient on ECMO with an impaired thrombophilia diagnostic, who got a new shunt after 24 h due to pulmonary thrombosis; the patient died one week after that due to disseminated thrombosis in the shunt and the pulmonary arteries.

No pleural or pericardial effusions were documented (24, 48, and 73 h after stenting), and saturation improved in all patients.

No hematoma or acute femoral artery damage after the intervention was documented.

### Thrombotic events and sudden death after shunt stenting

We reported one case with the in-stent thrombotic event and one case with resuscitation after a crying attack without evidence of in-stent thrombosis. Both patients were already mentioned above. The third case was a sudden death in a patient with HLHS and partial anomalous drainage of the right pulmonary veins at a higher level in the vena cava superior. He had Stage I palliation with a 4 mm shunt. The shunt was stented with a 4.5 mm stent due to a stenosis. Three months later, a new stent of 5 mm diameter was required to delay the Glenn operation due to atypical insertion of the pulmonary veins in the SVC. Sudden death happened at home 60 days after the discharge. The patient was on aspirin. An acute shunt stent obstruction with a thrombosis was suspected to be the cause of the death.

### Antiplatelet therapy

At discharge, one patient was on rivaroxaban due to thrombocytopenia; four were on Aspirin and clopidogrel (inclusive of patients with Magnesium stents), and the rest were on Aspirin (inclusive of the patient with two stents in shunt, in whom a sudden death was reported).

### Follow up

Twenty-six patients were operated on (Glenn in 19, biventricular repair in 5, and 1.5 repair in 1 patient). One patient was operated on with tricuspid reconstruction 40 days after stenting and died postoperatively. Four patients were unfit for Glenn due to severe hypoplastic pulmonary arteries and were referred to palliation; two of them died. A sudden death was documented in the first one 40 days after stenting, and the second one died due to septic shock 34 days after the intervention. The need for a surgical revision of the shunt was documented in 2 patients. One of them was on ECMO after shunt obstruction, which was revascularized and stented. Twenty-four hours after stenting, the shunt was revised due to an ECMO-related thrombotic event. The patient died 1 week after the operation. In the other patient, the 3.5 mm shunt was changed to a 4 mm shunt 2 weeks after shunt dilation due to inadequate saturation

## Discussion

Several studies and case reports about MBTS interventions and treatment of shunt thrombosis in infants and adults have been conducted in the past 2 decades due to their importance in improving pulmonary artery growth and saving lives in some life-threatening cases of shunt obstruction (6–9).

This study aims to review our technique for intervening in stenosed and obstructed shunts and the results of the interventions done in our center in the past 4 years. Ligon et al. (6) showed that 25% of MBTSs during interventions were not amenable to access from the femoral artery, and accessing the carotid artery was required in these patients to achieve the interventions with a lower procedure time. In contrast to the previous results, all intervened shunts in our current report were successfully accessed from the femoral artery without the need to access the carotid or axillary artery; in one case, the anastomosis between the shunt and the TBC led to an acute angle, which made crossing the shunt from the femoral artery difficult, but the shunt was accessed using three wires, which stabilized the advancement of the materials to the shunt (Figure 6). However, the intervention-related radiation time in our cohort, which included the intervention and the diagnostic performed in stable patients, was equal to or lower than that reported via carotid artery shunt interventions. Bonnet et al. (4) reported about 33 interventions in obstructed shunts over 20 years with 15% intervention-related complications, including one catheter-induced transient complete atrioventricular block, one cardiac tamponade, and one massive thrombo-embolic stroke. In contrast, in our report with 24 emergent interventions due to severe stenosis, including 4 with totally obstructed shunts, we have documented no intervention-related complications, including no intervention-related accessed vessel damage or occlusion (5). Our results are in agreement with a previous study (5) regarding the complete surgical resection of the stents implanted in the shunts at the time of elective shunt takedown.

Wells et al. (2) showed that most MBTSs showed evidence of stenosis by the time of takedown, and 21% have greater than 50% obstruction with histological evidence for myofibroblastic proliferation often associated with organized thrombosis, which can lead to obstruction. Dilatation and stenting of thrombotic shunts might lead to the embolization of thrombotic material into the pulmonary arteries (10). For this reason, good heparinization is required during and after the intervention to prevent against an acute thrombotic event in the shunt and the pulmonary arteries. In our cohort, severe thrombotic events in the shunt and the pulmonary arteries were difficult to avoid in patients with an impaired thrombophilia diagnostic and a previous history of multiple thrombotic events despite intensive intravenous anticoagulation and intensive control of the coagulation laboratory in the ICU. A thrombotic event, arrhythmias and septic shock were suspected to be the cause of sudden resuscitation in 1 patient and sudden death in another without reported evidence. Both were on aspirin, which seems to be ineffective alone in patients with more than one stent per shunt, and dual antiplatelet therapy should be considered in such cases (5).

### Lessons learned from our experience and conclusion

Interventions on stenosed and obstructed MBTS to save lives are feasible and could be performed without intervention-related complications or deaths when the procedure is well prepared with adequate knowledge of all materials and accessed routes needed for the interventions, which could facilitate the procedure and lead to satisfactory results.

We recommend performing a thrombophilia diagnostic in all patients who require shunts and shunt interventions to minimize thrombotic events by choosing a better anticoagulation strategy as early as possible.

Partial stenting of the shunt could lead to a new stenosis in the non-stented shunt tissue. In the case of localized shunt stenosis, the shunt should be completely stented to avoid new stenosis and the need for a second stent.

In some cases of atypical shunt anastomosis, the axillary and carotid accesses should be considered to facilitate the intervention and shorten the radiation and intervention time.

The need for shunt stenting with more than one stent or the need for reintervention seems to increase the risk of shunt thrombosis. Aspirin alone seems ineffective, and dual antiplatelet therapy is strongly recommended.

Based on our experience, the diameter of the 3.5 mm and 4 mm shunts could be enlarged via stent to 4.5 and 5 mm, respectively. This makes shunt stenting, in some cases, an alternative to surgical revision of the shunt to delay the subsequent operation when the patient is unfit for surgery or when there is a contraindication.

Avoiding diuretics and dehydration in patients with shunt stenting who are discharged home with effective antiplatelet therapy and closed echocardiographic controlled to exclude shunt stenosis is crucial to minimize the risk of sudden death related to shunt thrombosis.

## Data Availability

The original contributions presented in the study are included in the article/Supplementary Material, further inquiries can be directed to the corresponding author.
